# Aggressive angiomyxoma of pelvis: A case report and literature review

**DOI:** 10.1097/MD.0000000000031617

**Published:** 2022-11-18

**Authors:** Xue-Mei Lin, Li Wang, Qiong Wang

**Affiliations:** a Department of Pathology, Institute of Basic Medicine and Forensic Medicine, North Sichuan Medical College, Nanchong, Sichuan, China; b Department of Pathology, Affiliated Hospital of North Sichuan Medical College, Nanchong, Sichuan, China.

**Keywords:** aggressive angiomyxoma, angiomyofibroblastoma, clinicopathologic feature, mesenchymal tumor

## Abstract

Aggressive angiomyxoma (AAM) is a rare mesenchymal tumor primarily involving the lower genital tract of reproductive females. It often shares pathologic morphology with other mesenchymal lesions, which result in diagnostic difficulties for pathologists.

**Patient concerns and diagnoses:**

We described the case of a 32-year-old female presenting with a pelvic mass. Imaging examination showed a “swirling sign” within the mass. The mass was 10.2 × 10 × 7.7 cm, located in the right front of the uterus, with unclear demarcation from the surrounding organs and tissues. The gross appearance was grayish brown with a solid section and a myxedematous cut surface. Microscopically, it was a mesenchymal tumor with a presence of perivascular smooth muscle fibers radiating from the blood vessel and an infiltrative growth pattern. The pelvic AAM was diagnosed based on clinicopathologic and imaging features.

**Interventions and outcomes:**

A surgery with local excision of the mass was performed. The patient experienced 1 relapse during 2-year follow-up and underwent the radiation therapy.

**Lessons:**

When the pathological morphology of AAM overlaps with other mesenchymal lesions, the comprehensive understanding of tumor clinicopathological characteristics combined with imaging features is important for the accurate diagnosis of AAM.

## 1. Introduction

Aggressive angiomyxoma (AAM) is a rare mesenchymal tumor that primarily involves the female lower genital tract during reproductive period, with the characteristics of local infiltration, local recurrence and distant metastasis.^[1]^ The clinicopathological features of AAM partially overlap with other mesenchymal lesions, leading to diagnostic difficulties for pathologists.^[2–4]^ In the present case, the morphological features overlap with those of angiomyofibroblastoma (AMF). Our report highlights the importance of the clinicopathological and imaging features in the diagnosis of AAM.

## 2. Case report

**Figure 1.**
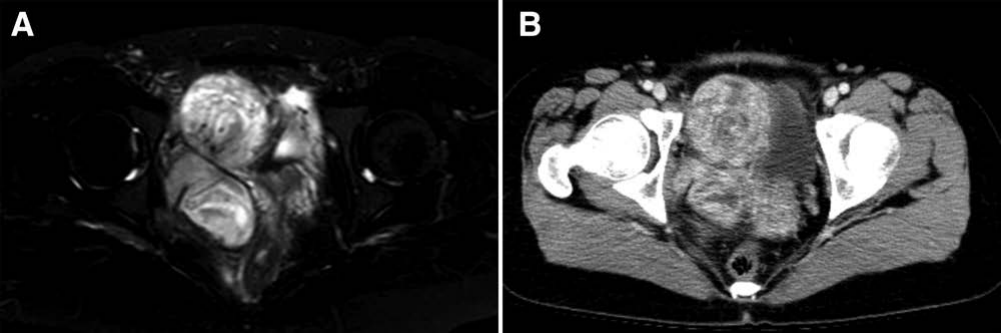
Imaging findings of aggressive angiomyxoma. The characteristic “swirling sign” within the mass was revealed by enhanced computed tomography scans (A) and magnetic resonance imaging (B).

In March 2020, a 32-year-old woman presented to our hospital with a pelvic mass on routine ultrasound examination. By enhanced computed tomography and enhanced magnetic resonance imaging, the mass size was 10.2 × 10 × 7.7 cm, located in the right front of the uterus, with unclear demarcation from the right vaginal wall, right levator ani muscle and obturator muscles. A “swirling” characteristic was observed within the mass (Fig. 1).

Ultrasound-guided puncture of the mass was conducted. Histological examination revealed the lesion was composed of sparsely arranged spindle-shaped and stellate-shaped cells, without obvious atypia and mitosis. The myxoid stroma was interspersed with several thin-walled vessels. The cells were positive for CD99, and negative for cytokeratin, calretinin, epithelial membrane antigen, CD34 and signal transducer and activator of transcription 6. Finally, the lesion was diagnosed as a mesenchymal tumor, but the specific type was not identified. Then, a local excision of the tumor was performed.

**Figure 2.**
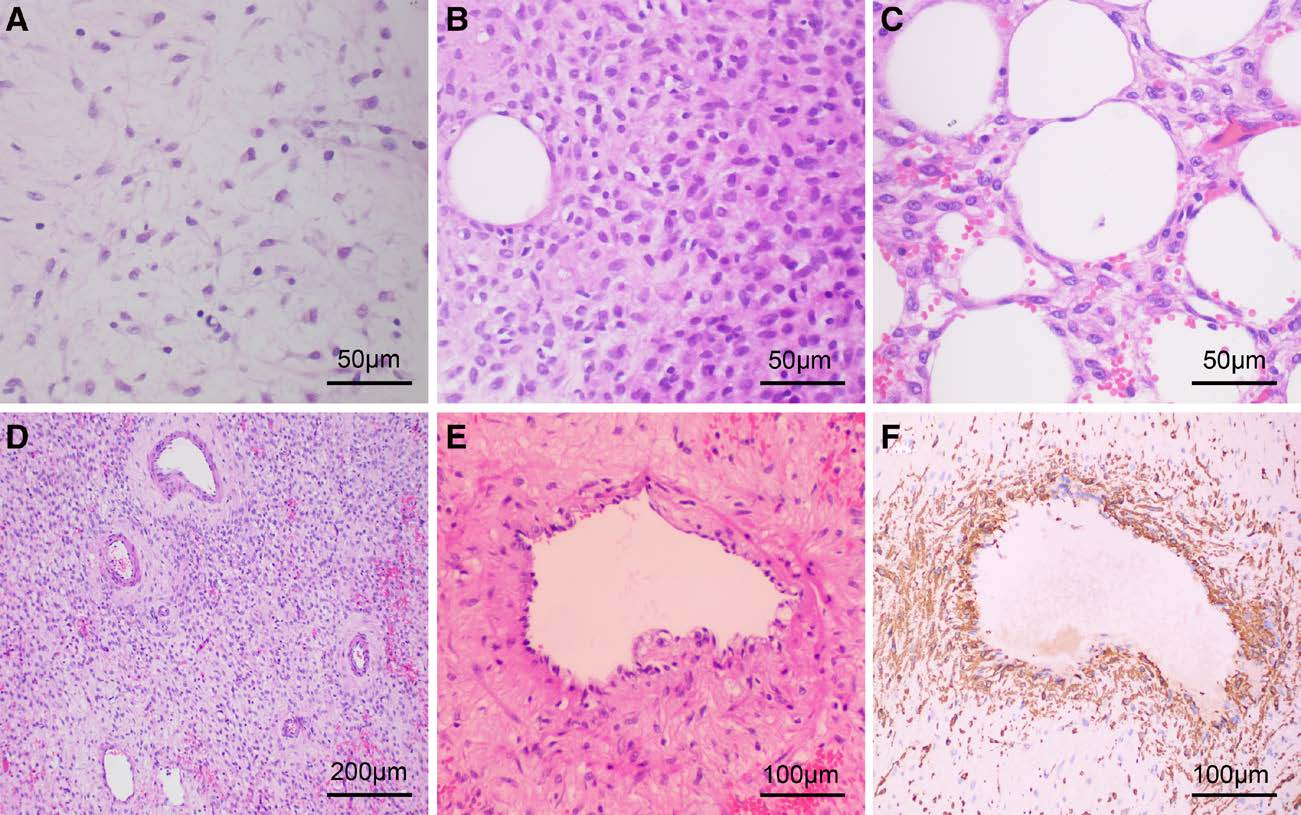
The microscopic features of aggressive angiomyxoma. Microscopical examination revealed a tumor of alternating hypocellular (A) and hypercellular (B), infiltrating the surrounding adipose tissue (C). The tumor cells were oval- and spindled-shaped with pale to eosinophilic cytoplasm, without cellular atypia and mitotic activity (B). The myxoid stroma was interspersed with many disorganized blood vessels, ranging in size from thin-walled capillaries and venules to thicken-walled blood vessels (D). A small number of smooth muscles differentiated cell bundles were present around blood vessels (E) and positive for SMA (F). (A, 400 × HE; B, 400 × HE; C, 400 × HE; D, 100 × HE; E, 200 × HE; F, 200 × immunohistochemistry). HE = hematoxylin and eosin.

The gross appearance of the mass after operation was grayish brown with a solid section, and displayed a myxedematous cut surface. It measured 9.5 × 8 × 6 cm in the maximum dimension. Microscopical examination revealed a tumor of alternating hypocellular and hypercellular (Fig. 2A and B), infiltrating the surrounding adipose tissue (Fig. 2C). The tumor cells were oval-shaped and spindled-shaped with pale to eosinophilic cytoplasm (Fig. 2B), without cellular atypia and mitotic activity. The myxoid stroma was interspersed with many disorganized blood vessels, ranging in size from thin-walled capillaries and venules to thicken-walled blood vessels (Fig. 2D). A small number of smooth muscles differentiated cell bundles were present around blood vessels (Fig. 2E).

The tumor cells were diffuse positive for estrogen receptor (ER), progesterone receptor (PR), desmin, vimentin and CD99, focally positive for CD34 and B-cell leukemia/lymphoma 2 gene, and negative for S-100, murine double minute 2, P53, signal transducer and activator of transcription 6, epithelial membrane antigen, inhibin, calretenin, human melanoma black 45, CD10 and H-caldesmon. Smooth muscle actin (SMA) was positive for the smooth muscle differentiated cell bundles around blood vessels but negative for the muscular layer of blood vessels (Fig. 2F). The cell proliferation marker Ki67 accounted for about 2% of total cells. The tumor was finally diagnosed as AAM.

The patient experienced 1 recurrence during the 2-year follow-up and underwent the radiation therapy. At present, the patient recovered well.

## 3. Discussion

AAM was firstly reported by Steeper and Rosai in 1983,^[1]^ and the term aggressive was used to emphasize the infiltrative and local recurrent nature of the tumor. As AAM is a rare tumor, the PubMed database was searched and more than 500 cases of approximate 300 available articles were retrieved from 1983 to 2020. Based on these articles, the histogenesis of AAM was still unknown. In 1990, Koichi Mandai et al^[5]^ firstly proposed that AAM may originate from primitive pluripotent stem cells. In 1997, Silverman et al^[6]^ proposed that some AAM may originate from the dendritic cells of CD34 + in the pelvic genital tract. Additionally, partial pathologists speculated that AAM may originate from fibroblasts or myofibroblasts based on the pathological and ultrastructural characteristics of the tumor cells. In a word, only a few literature attempted to investigate the histogenesis of AAM and no consensus was achieved.

According to the accumulated case reports, fluorescence in situ hybridization analysis demonstrates that 12q15 chromosomal aberration leads to a rearrangement of high mobility group protein A2 gene in almost half of AAM. However, the role of high mobility group protein A2 gene in the occurrence and development of AAM is elusive. Moreover, the tumor cells are positive for ER, PR and androgen receptor (AR), which suggest that hormones may manipulate the occurrence and/or development of AAM.^[7,8]^

At present, the clinical characteristics of AAM have been well documented. AAM mainly involves the lower genital tract of reproductive women, with a peak incidence in the fourth decade of life. The ratio of male to female is 1:6.6.^[9]^ Patients often present with a mass, with or without compressive symptoms and the manifestations are not specific.

Surgical resection is the main treatment for AAM, and gonadotropin releasing hormone agonists have also been used as adjuvant treatment. Gonadotropin releasing hormone agonists can reduce the tumor volume before operation,^[10]^ prevent the recurrence of the tumor^[11]^ and also treat the residual and/or recurrent tumor.^[12–15]^ The reported recurrence rate varied from 35% to 72% and the relapse usually occurred in the first 5 years after surgery.^[9]^ The longest recurrence time reported in the literature is 20 years.^[16]^ There are even reports of lung metastasis^[17,18]^ and death^[19]^ in patients. Therefore, patients need long-term follow-up after operation.

The formulation and implementation of an effective therapeutic schedule for AAM patients are based on the accurate pathological diagnosis of the mass. However, the pathological morphology of AAM may resemble that of other mesenchymal lesions, leading to diagnostic difficulties for pathologists. The mass of AAM described in the literature is usually deep and typically poorly demarcated, ranging from 1 to 60 cm in diameter. Microscopically, the tumor is composed of myxoid stroma interspersed with numerous blood vessels of different calibers and types. The tumor cells are sparsely and uniformly distributed, with spindle and/or stellate shapes, without mitotic activity and nuclear atypia. Delicate smooth muscle bundles are often seen adjacent to blood vessels, which are considered a characteristic feature of AAM.^[2,20]^ The tumor cells had variable expressions of vimentin, desmin, CD34, SMA, ER and PR, but these markers are not specific for the pathological diagnosis of AAM.

In the present case, the mass was located in the pelvis, showing a grayish brown appearance and a myxedematous cut surface. The microscopic feature was characterized by the presence of perivascular smooth muscle fibers radiating from the blood vessel and the infiltrative growth pattern. These findings were consistent with those reported in previous literature. However, the microscopic appearance was of some interest beyond those described in the literature. The tumor cells showed areas of hypocellularity and hypercellularity with oval- and spindle-shaped nuclei, pale and/or eosinophilic cytoplasm, overlapping with AMF. Therefore, the distinction between AAM and AMF is the focus of pathological diagnosis.

In contrast to AAM, AMF usually involves the vulva, and the mass is usually less than 5 cm in diameter. Microscopically, AMF has a well-defined boundary, and the typical feature of AMF is plump spindled- and epithelioid-appearing cells arranged in corded and nested patterns and commonly concentrated around blood vessels. In our case, the mass was located in the pelvis, infiltrating the surrounding adipose tissue. Furthermore, smooth muscle bundles, as a pathological diagnostic feature of AAM, were seen adjacent to blood vessels. These clinicopathologic features were consistent with that AAM rather than AMF. Additionally, a large pelvic mass with abdominal/perineal extension in reproductive age female patient should lead to suspicion of AAM,^[21]^ and a characteristic “whirlpool sign” within the mass is considered to be a distinctive diagnostic feature of AAM.^[22]^ In our case, the mass extended into perineum and there was a characteristic “whirling sign” within the mass. These imaging features were consistent with AAM but not AMF. Finally, the tumor was diagnosed as AAM.

In addition to AMF, AAM also needs to be differentiated from other mesenchymal lesions that occur in the lower genital tract, including fibroepithelial stromal polyp, cellular angiofibroma, superficial cervicovaginal myofibroblastoma, and superficial angiomyxoma. The clinical and pathological features of mesenchymal lesions in the female lower genital tract are summarized in Table 1.

**Table 1:** Comparison of clinical and pathological features among mesenchymal lesions.

	AAM	Angiomyofibroblastoma	Fibroepithelial stromal polyps
Sites	Deep location	Superficial location	Superficial location
Local recurrence	29.0% rate	Occasionally	Rarely
Boundary	Poorly circumscribed	Well circumscribed	Poorly circumscribed
Tumor size	1–60 cm	<5 cm	Varying size
Morphology	Spindle/stellate shaped	Ovoid, spindle, epithelioid, plasmacytoid	Spindle/stellate shaped
Cellularity	Low cellularity	Alternating hypocellular and hypercellular area	Low cellularity
Stroma	Myxoid stroma	Fibrous but focally may be myxoid	Fibrous, edematous or somewhat myxoid,
Mitotic activity	Absent	Rare or absent	Frequent
Blood vessels	Thin-walled, capillary-like vessels to large vessels	Thin-walled, capillary-like vessels	Thin-walled vessels
Immunophenotype	Positive: vimentin, ER, PR	Positive: vimentin, ER, PR, desmin	Positive: vimentin, ER, PR, desmin
	Negative: S-100	Negative: S-100	Negative: S-100
	Variable expression: SMA, desmin, CD34	Variable expression: SMA, CD34	Variable expression: SMA, CD34
	**Superficial angiomyxoma**	**Cellular angiofibroma**	**Superficial cervical-vaginal myofibroblastoma**
Sites	Superficial location	Superficial location	Superficial location
Local recurrence	30%–40% rate	Occasionally	Uncommonly
Boundary	Poorly circumscribed	Well circumscribed	Well circumscribed
Tumor size	<5 cm	<5 cm	1–6.5 cm
Morphology	Spindle/stellate shaped	Spindle shaped	Ovoid, spindle or stellate shaped
Cellularity	Low cellularity	Moderate cellularity	Varying cellularity
Stroma	Abundant myxoid stroma	Fibrous stroma	Collagenous stroma
Mitotic activity	Few or none	Few, occasionally easily identified	Few or none
Blood vessels	Thin-walled, capillary-like vessels	Small to medium size vessels	Capillaries to thick-walled vessels
Immunophenotype	Positive: vimentin, CD34, SMA	Positive: vimentin, ER, PR	Positive: vimentin, desmin
	Negative: ER, PR, desmin,	Negative: SMA, desmin, h-caldesmon	Negative: S-100, H-caldesmon
	Variable expression: S-100	Variable expression: CD34, P16	Variable expression: CD34, CD99, SMA, ER, PR

AAM = aggressive angiomyxoma, ER = estrogen receptor, PR = progesterone receptor, SMA = smooth muscle actin.

## 4. Conclusion

In conclusion, AAM is a rare mesenchymal tumor characterized by local infiltration, recurrence, distant metastasis and even death. Patients with AAM need long-term follow-up after surgery. AAM may share histological features with other mesenchymal lesions, resulting in diagnostic difficulties for pathologists. A comprehensive understanding of tumor clinicopathological characteristics combined with imaging features is important for the accurate diagnosis of AAM.

## Author contributions

**Conceptualization:** Qiong Wang.

**Data curation:** Xue-Mei Lin, Li Wang, Qiong Wang.

**Formal analysis:** Xue-Mei Lin, Qiong Wang.

**Investigation:** Xue-Mei Lin, Li Wang.

**Methodology:** Xue-Mei Lin, Li Wang.

**Project administration:** Qiong Wang.

**Supervision:** Qiong Wang.

**Writing – original draft:** Xue-Mei Lin.

**Writing – review & editing:** Xue-Mei Lin, Li Wang, Qiong Wang.
